# 3C-SiC Nanowires In-Situ Modified Carbon/Carbon Composites and Their Effect on Mechanical and Thermal Properties

**DOI:** 10.3390/nano8110894

**Published:** 2018-11-01

**Authors:** Hongjiao Lin, Hejun Li, Qingliang Shen, Xiaohong Shi, Tao Feng, Lingjun Guo

**Affiliations:** 1 State Key Laboratory of Solidification Processing, Northwestern Polytechnical University, Xi’an 710072, China; lin.hong.jiao@163.com (H.L.); shenqingliang@mail.nwpu.edu.cn (Q.S.); fengtao@nwpu.edu.cn (T.F.); guolingjun@nwpu.edu.cn (L.G.); 2 School of Mechanics, Civil Engineering & Architecture, Northwestern Polytechnical University, Xi’an 710072, China

**Keywords:** SiC nanowires, C/C composites, in-situ growth, mechanical properties

## Abstract

An in-situ, catalyst-free method for synthesizing 3C-SiC ceramic nanowires (SiCNWs) inside carbon–carbon (C/C) composites was successfully achieved. Obtained samples in different stages were characterized by X-ray diffraction (XRD), scanning electron microscopy (SEM), and Raman scattering spectroscopy. Results demonstrated that the combination of sol-gel impregnation and carbothermal reduction was an efficient method for in-situ SiCNW synthesis, inside C/C composites. Thermal properties and mechanical behaviors—including out-of-plane and in-plane compressive strengths, as well as interlaminar shear strength (ILLS) of SiCNW modified C/C composites—were investigated. By introducing SiCNWs, the initial oxidation temperature of C/C was increased remarkably. Meanwhile, out-of-plane and in-plane compressive strengths, as well as interlaminar shear strength (ILLS) of C/C composites were increased by 249.3%, 109.2%, and 190.0%, respectively. This significant improvement resulted from simultaneous reinforcement between the fiber/matrix (F/M) and matrix/matrix (M/M) interfaces, based on analysis of the fracture mechanism.

## 1. Introduction

Carbon–carbon (C/C) composites, composed of carbon fibers and a carbon matrix, are important thermal-structural materials. C/C composites can retain their strength and stiffness under ultra-high temperatures, and they exhibit unique thermal properties, such as high heat capacity, high thermal conductivity, low thermal expansion, and good thermal shock resistance. Therefore, C/C composites are widely applied in aerospace engineering and used in industrial devices [1,2,3]. Mechanical and thermal properties of C/C composites mainly depend on their carbon microstructures. Generally, the carbon matrix is deposited inside the carbon fiber preform by chemical vapor infiltration (CVI), to form C/C composites. In addition, the process of infiltrating the carbon matrix greatly affects the interface in yielded composites. Bridge interfaces for both stress and heat transfer significantly impact the mechanical and thermal performance of C/C composites [4,5,6], as well as impact the mechanical and thermal performances of C/C composites.

Mechanical and thermal properties are key performance indicators for assessing high-temperature structure materials [7]. As for C/C composites, mechanical and thermal properties mainly depend on the microstructure of the carbon materials. An interface is the bridge, through which load and heat can be transferred in composites [8,9]. There are mainly two kinds of interfaces in C/C composites: fiber/matrix (F/M) interfaces—interfaces between the fiber and its surrounding matrix—and matrix/matrix (M/M) interfaces, which result from the layered feature of the matrix and refers to interfaces between the layered matrix. The poor bonding strength of both interfaces leads to low interlaminar shear strength and compressive strength of the composites. In recent years, numerous investigations have been carried out to modify these interfaces, in order to improve both the mechanical and thermal performance of C/C composites [10,11,12].

Silicon carbide nanowires (SiCNWs) have attracted much interest because of their excellent elasticity and strength, which are much better than those of SiC ceramic bulks and whiskers. Therefore, SiCNWs have been proven to have outstanding secondary reinforcing phases in ceramic, metals and polymer matrix composites [13,14,15,16]. However, the application of SiCNWs inside of C/C composites has been rarely studied. Up to now, the most commonly applied method employed has been the well-known, vapor-liquid-solid (VLS) growth mechanism, which usually uses a metal as the catalyst, due to its unique properties [17]. However, it is very difficult to remove the residual metal catalyst from SiC nanowires and it always acts as a contamination, resulting in the degradation of their electronic and mechanical properties, under harsh environmental conditions [18,19]. Especially when SiC nanowires are applied as the reinforced phase in C/C composites at high temperatures, the metal catalyst will cause devastating side effects. Catalysts can accelerate the oxidation process, which can corrode fibers. At the same time, because of the complexity of the internal structure of C/C composites, it is difficult to perform in-situ synthesis of nanowires. Hence, a greater enhancement of the embedded reinforcement phases in C/C composites should be carried out. Technological challenges affecting C/C composites are summarized below: (a) metal catalysts should be avoided; (b) a porous material with as many growth spaces as the growth medium is needed; (c) the reinforcement phase should be dispersed uniformly inside the matrix.

In the present work, we demonstrated a feasible way to achieve in-situ, catalyst-free growth of SiCNWs in C/C composites. Porous carbon felt was used as the growth medium, whereas silica xerogel and pyrolytic carbon were used as the source materials. Finally, SiCNW-C/C composites were obtained through densification, using the TGCVI (Thermal Gradient Chemical Vapor Infiltration) method. The morphology, crystal microstructure, composition, and growth mechanism of SiCNWs were studied. As for the SiCNW-C/C composites, mechanical properties—including out-of-plane and in-plane compressive strengths, as well as interlaminar shear strength (ILLS), were also tested. Furthermore, we analyzed the reinforcement mechanism of C/C composites with SEM images of the fracture surfaces, which demonstrated the excellent mechanical properties of SiCNWs during the reinforcement phase.

## 2. Experimental Section

### 2.1. Preparation of SiCNW-C/C Composites

**Figure 1:**
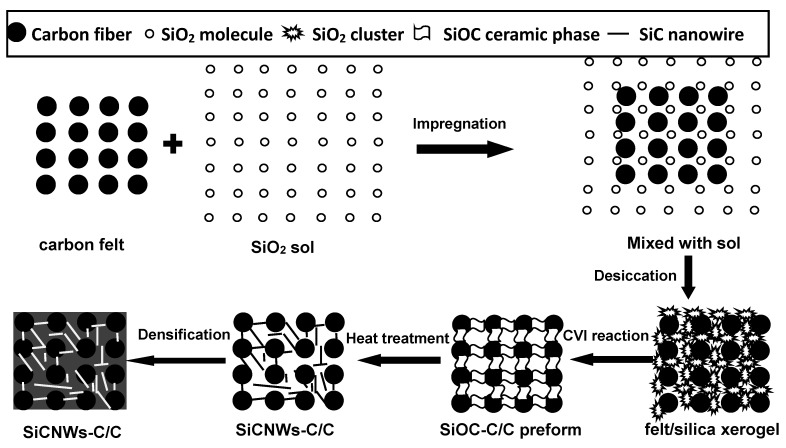
Schematic diagram of the formation of silicon carbide nanowires with carbon-carbon (SiCNW-C/C) composites. CVI = chemical vapor infiltration.

Commercially available tetraethoxysilane (TEOS), ethanol absolute (EtOH, AR; Hongyan Reagent Factory, Tianjin, China), deionized water (H_2_O, AR), hydrochloric acid (HCl, AR; Hongyan Reagent Factory, Tianjin, China), and 2D needled carbon felt (density: 0.54–0.56 g/cm^3^, fiber diameter: 6–7 μm; Tianniao, Jiangsu, China) were used as raw materials. SiO_2_ sol was prepared by the method reported in our previous work [20]. Figure 1 shows the schematic illustration for the fabrication of C/C composites, with in-situ grown SiCNWs. Firstly, 2D needled carbon fiber felts (120 mm × 80 mm × 70 mm) were impregnated with SiO_2_ sol to obtain carbon felt/silica xerogel. Then the carbon felt/silica xerogel was placed at the center of a tube furnace. The heating rate was 10 °C/min and the flow rate of Ar gas was controlled at 1.5–1.6 L/h. When the temperature reached 1050 °C, it was maintained for 2 h. After that, the furnace was naturally cooled to room temperature and SiOC-C/C composite preforms were obtained through the ICVI (Isothermal Chemical Vapor Infiltration) reaction process.

**Figure 2:**
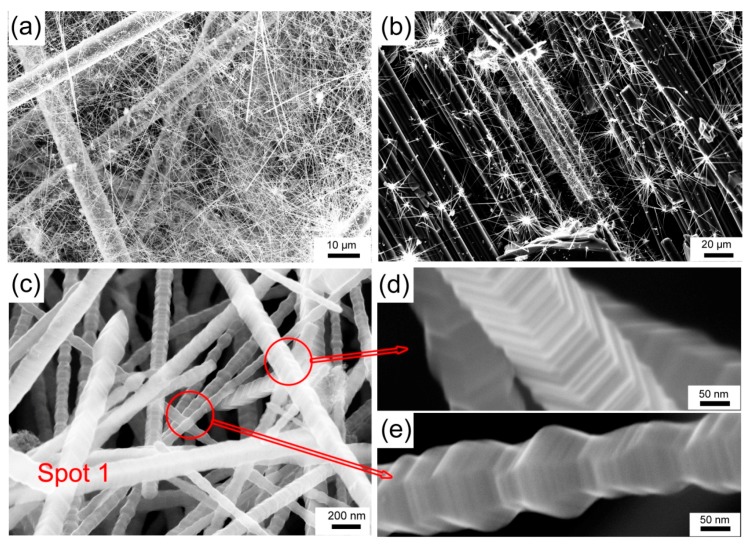

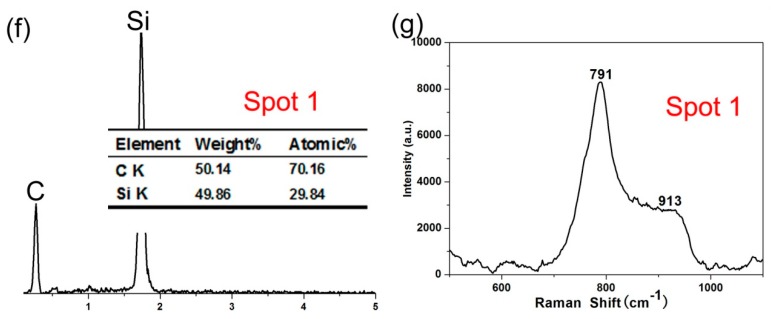
(**a**) Low-magnification of the needling felt layer, (**b**) low-magnification of the non-woven layer, (**c**) high-magnification, (**d**) columnar, and (**e**) beaded SEM images of the SiCNWs, (**f**) EDS of the SiCNWs, (**g**) and Raman spectra of the SiCNWs.

In order to obtain the SiCNWs, SiOC-C/C preforms were heat treated in a graphite furnace, at 1550 °C for 2 h, under an Ar atmosphere and with a steady heating rate of 20 °C/min. After the heat treatment, the SiOC was converted into many cross-linked SiC nanowires, as shown in Figure 2. The last step was the densification of the preforms by TGCVI, with natural gas as the carbon source, nitrogen as the dilute, and a carrier gas at 950 °C. After 140 h of densification, the final density of the SiCNW-C/C composite was about 1.80 g/cm^3^.

### 2.2. Characterization

Scanning electron microscopy (SEM) images were taken on a field emission scanning electron microscopy (FESEM, ZEISS-SUPRA 55, Jena, Germany), equipped with energy dispersive X-ray spectroscopy (EDS). The chemical composition and phase identification of the synthesized felt/silica xerogel, SiOC-C/C preform, and SiOC-C/C and SiCNW-C/C composites were analyzed using Raman scattering spectroscopy (Raman, Renishaw inVia, Gloucestershire, England) and X-ray diffraction (XRD; PANalytical X’Pert Pro, Almelo, The Netherlands), with Cu Kα radiation (λ = 1.5418°A).

### 2.3. Test of Mechanical Properties

Differential thermal analysis and thermal gravimetry were carried out on a TGA/SDTA851 thermoanalyzer (Mettler Toledo, Zurich, Switzerland), within an air atmosphere, at a heating rate of 10 °C/min. Compressive strength (out-of-plane and in-of-plane, ASTM D3410) and interlaminar shear strength (ILSS; ASTM D5379)—as well as the compressive shearing of the composites before and after modification by the SiCNWs—were tested to demonstrate the effect of SiCNWs in increasing the C/C composite’s mechanical properties. The samples prepared for tests, with a loading rate of 0.5 mm/min at room temperature, were placed in square grids with the size of 10 mm × 10 mm × 10 mm. All these samples were tested, using an electronic universal testing machine (Shenzhen SANS Testing Machine Co., Ltd., SANS CMT5304-30, Shenzhen, China). Final results were achieved by computing means of at least five samples. Both the compressive strength and shear strength were calculated by *σ* = *P*/*S*, where *σ* means the calculated mechanical strength, *P* denotes the compressive force, and *S* signifies the area of force.

## 3. Results and Discussion

### 3.1. Morphology of SiCNWs

The as-obtained SiCNWs were characterized by SEM to identify their morphology. The non-woven layer and needling felt layer of the SiCNW-C/C preform could be observed from the low magnification SEM image of the SiCNWs. As shown in Figure 2a,b, there was a large number of nanowires randomly distributed on the surface of the carbon fibers, whose lengths varied from several to hundreds of micrometers. Compared to the needling felt layer, SiCNWs in the non-woven layer were relatively few and radiated from the carbon fiber to the surrounding stretch. This was because there was more reaction space and Si source in the needling felt layer. Figure 2c indicates that mainly two types of SiCNWs existed. Corresponding high-magnification SEM images in Figure 2d,e clearly reveal the shape of the nanowires, with a diameter of approximately 100–200 nm, and were columnar and beaded, respectively. The specific elemental composition of the as-prepared sample, taken from Spot 1 (in red) in Figure 2c was further investigated using EDS, as displayed in the inset in Figure 2f. Due to the influence of the carbon matrix, the atomic ratio of Si and C was about 1:3. The Raman spectra (Figure 2g) show a peak at around 791 cm^−1^ and a broad peak in Spot 1 (in red), which were attributed to the 3C-SiC nanowires [21].

### 3.2. Phase and Structure Characterization

**Figure 3:**
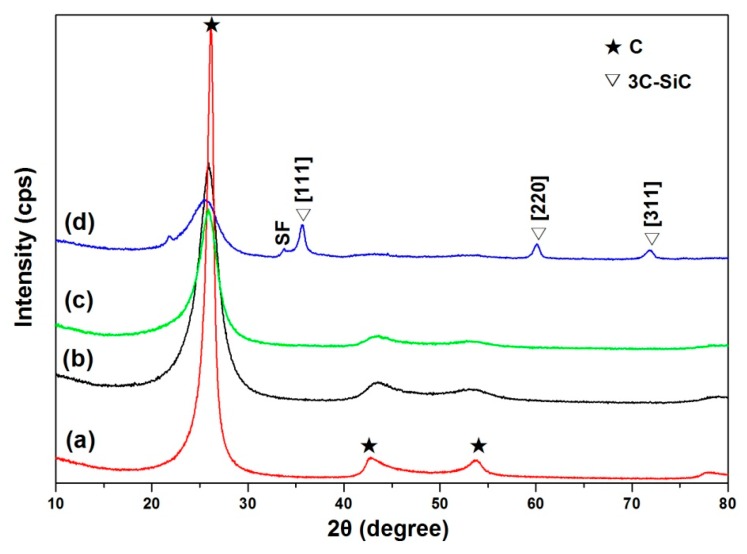
X-ray diffraction patterns for composites at different stages: (**a**) The carbon felt, (**b**) felt/silica xerogel, (**c**) SiOC-C/C preform, and (**d**) SiCNW-C/C perform.

Figure 3a–c shows the X-ray diffraction pattern of the carbon fiber felt, felt/silica xerogel, and SiOC-C/C preform. One main symmetrical diffraction peak at 25.9° was observed, which was assigned to the diffraction plane (002) of the sp^2^ carbon materials [22,23]. Figure 3d shows the diffraction pattern of the fiber felt, after the growth of the SiCNWs. It is characterized by the extra four diffraction peaks, in comparison to the other samples. Intensities of the main peaks at 35.7°, 60.1°, and 71.2° all match the 3C-SiC nanowires (JCPDS Card no. 29-1129) which were attributed to the diffraction of the <beta>-SiC (111), (220), and (311) planes [24]. Additionally, the low-intensity peak (SF) at 33.48° on the left shoulder of (111) peak was typically observed in the XRD spectra of the 3C-SiC nanowires, which was usually ascribed to stacking faults within the crystals [25].

**Figure 4:**
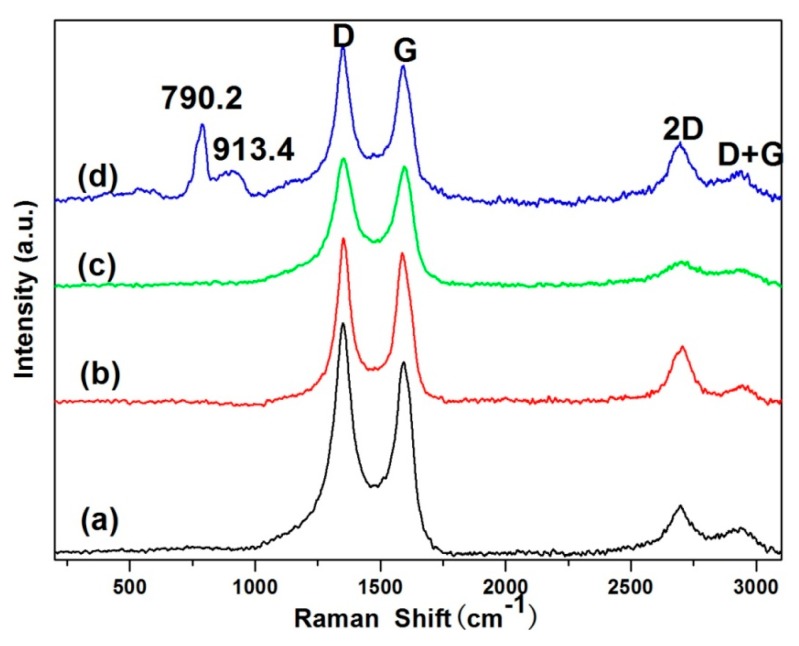
Raman spectra for composites at different stages: (**a**) The carbon felt, (**b**) felt/silica xerogel, (**c**) SiOC-C/C preform, and (**d**) SiCNW-C/C perform.

Typical Raman spectra of specimens at different stages are shown in Figure 4. There were four spectra of different stage samples which contained: Carbon felt (a), felt/silica xerogel (b), the SiOC-C/C preform (c), and (d) the SiCNW-C/C preform. The bands located near 1340 cm^−1^ (D band), 1580 cm^−1^ (G band), 2690cm^−1^ (2D band), and 2930 cm^−1^ (D+G band) can be clearly observed in the four spectra. According to previous research [26,27], the peak at 1340 cm^−1^ and 1580 cm^−1^ are usually associated with in-plane vibrations and in all sp^2^ bonded carbon atoms. The weak peak near 2690 cm^−1^ was due to the overtone of the D band and the broad peak, at about 2930 cm^−1^, was attributed to the sum of the D and G bands. A peak at around 790 cm^−1^ and a broad peak from 914–970 cm^−1^ were observed in Figure 4d, which were attributed to the zone center transverse optical (TO) phonon modes and the longitudinal optic (LO) phonon mode of the 3C-SiC nanowires [28]. We noted that the LO (G) phonon line showed a low number compared to other SiC materials [29,30]. One possible reason for this broadening was the size confinement effect: When the size of a crystal is reduced to a nano scale—such as in nanowires—phonons can be confined in the space by crystal boundaries or defects. As it is the reactive material source of SiC, SiO_2_ may also have led to a widening of the Raman signals [31].

### 3.3. Reactivity of SiCNWs

**Scheme 1:**
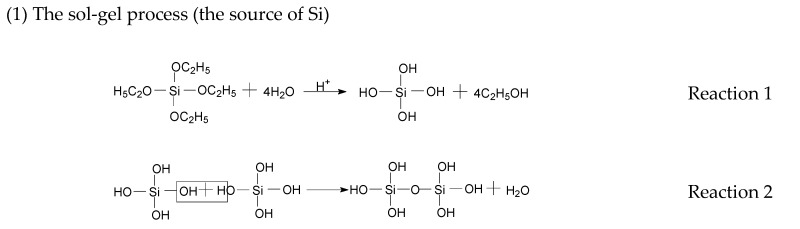

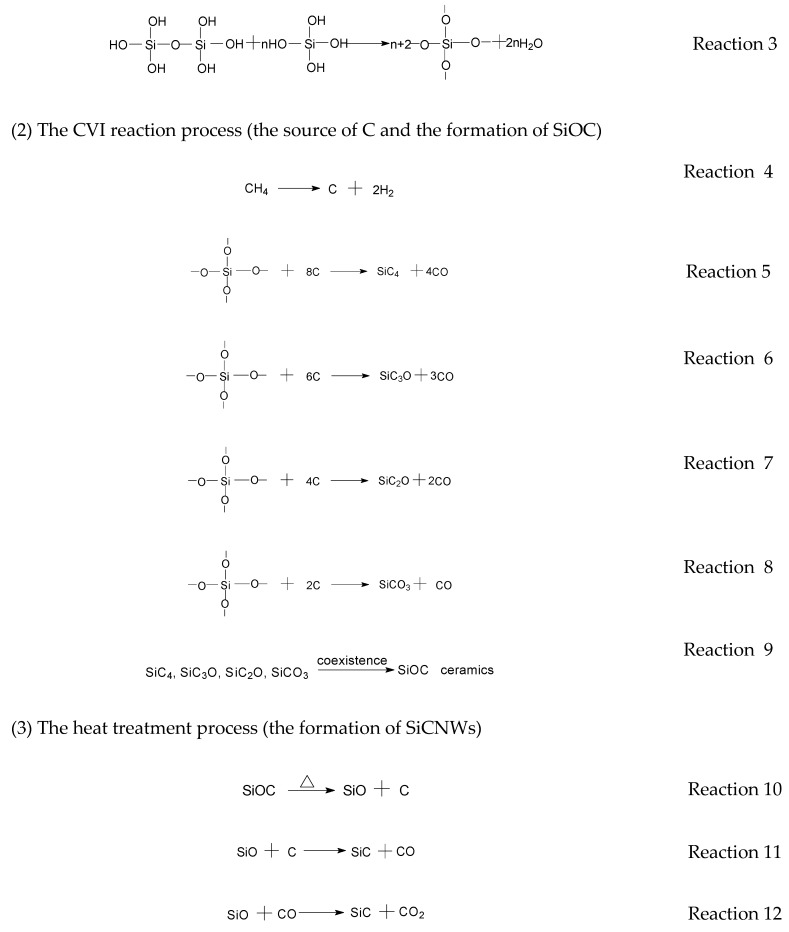
The possible reaction mechanism.

On the basis of the experimental results above, we propose a possible mechanism for the sol-gel process, the CVI reaction process, and the heat treatment process, shown in Scheme 1. In the sol-gel process, TEOS was firstly hydrolyzed into silanol (Reaction 1), then two silanol molecules lost one H_2_O molecule due to an elimination reaction (Reaction 2) [32,33]. Finally, (SiO_4_)_x_ could be obtained because of the interaction between the silanol molecules (Reaction 3). In the CVI reaction process, C vapor was obtained from decomposing CH_4_, as indicated in Reaction 4. When the C vapor seeped into the porous (SiO_4_)_x_, Reactions 5, 6, 7, and 8 could occur, from the view point of thermodynamics. After that, the cross-linking reactions [34] between products of SiC_4_, Si_3_O, SiC_2_O_2_, and SiCO_3_, reacted. Finally, the porous SiOC material could be received. As no catalyst was used during the preparing procedure, no droplets were observed at the tips or over the surfaces of the SiCNWs by the SEM and TEM characterizations. Therefore, we propose a catalyst-free vs. growth mechanism for the growth of SiCNWs [25,35,36]. As shown in the heat treatment process, thermal SiOC can readily decompose into free SiO groups and C (Reaction 10). When the pressure of SiO and C vapor was elevated to supersaturating conditions, the carbothermal reduction reaction involving two key reactions (Reactions 11 and 12) occurred inside the carbon felt, which led to the growth of SiCNWs. The initial SiC embryos could be formed by heterogeneous nucleation of the SiO gas with carbon particles, according to Reaction 11. As SiO and CO deposited on the tip of the growing SiC embryos, growth of SiCNWs gradually proceeded by the gas-phase reaction (Reaction 12) [23]. Another important aspect was that the porous characteristic of carbon felt and SiOC not only provided a wide growth space but also increased the reaction area.

### 3.4. Thermal Analysis

**Figure 5:**
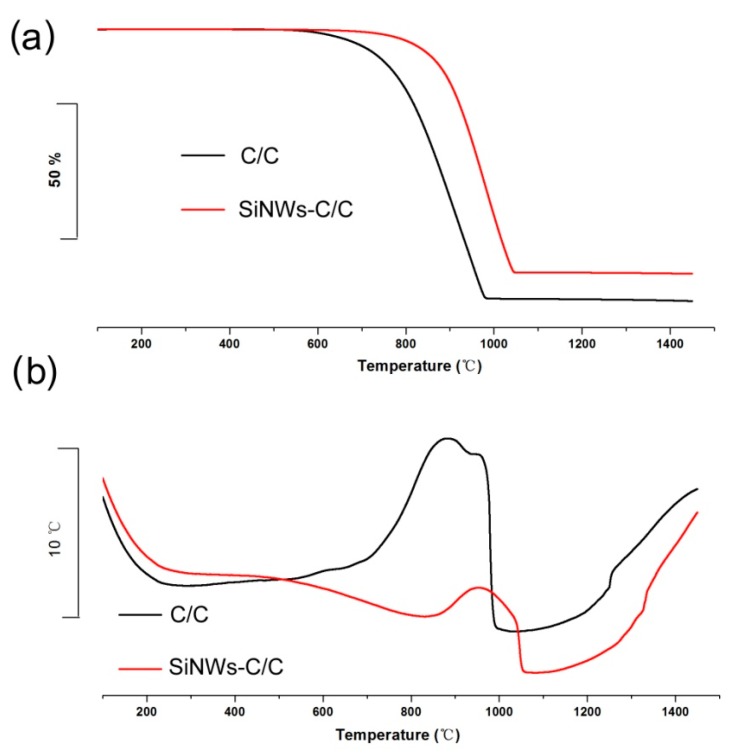
TGA (thermo-gravimetric analysis) (**a**) and SDTA (synchronous differential thermal analysis) (**b**) curves of the C/C and SiCNW-C/C composite samples.

TGA (thermo-gravimetric analysis) and SDTA (synchronous differential thermal analysis) curves of the C/C and SiCNW-C/C composites—shown in Figure 5a,b—were determined to investigate thermal stability. From Figure 5a, it can be clearly seen that initial oxidation decomposition temperatures of SiCNW-C/C composites were higher than those of pure C/C, whose oxidation started at 750 °C and ended at 1050 °C. Compared with C/C composites, the initial and end temperatures differed by about 150 °C and 90 °C. Undecomposed parts of the SiCNW-C/C and C/C composites were 15% and 0%, respectively, meaning C/C composites were nearly completely oxidized and SiCNW-C/C composites were not. From the SDTA curves, shown in Figure 5b, both C/C and SiCNW-C/C composites had obvious exothermic peaks, but the heat released from the C/C oxidation reaction was significantly more than that of the SiCNW-C/C composite. Therefore, the addition of SiCNWs could decrease the heat of the reaction to some extent.

### 3.5. Mechanical Properties

**Figure 6:**
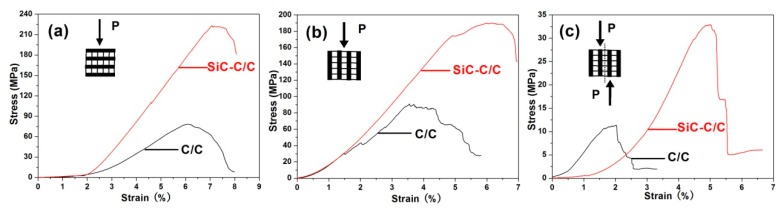
Mechanical properties of C/C and SiCNW-C/C composites: (**a**) Out-of-plane and (**b**) in-plane compressive strengths and (**c**) interlaminar shear strength (ILSS).

As shown in Figure 6 and Table 1, mechanical property tests indicated that SiCNW-C/C composites had significant improvements, including out-of-plane and in-plane compressive strengths, as well as interlaminar shear strength (ILSS) of 227%, 109%, and 190%, respectively. Compared with C/C composites, SiCNW-C/C composites also had 49% and 111% improvements in the out-of-plane and in-plane compressive moduli.

### 3.6. Fracture Surfaces

**Figure 7:**
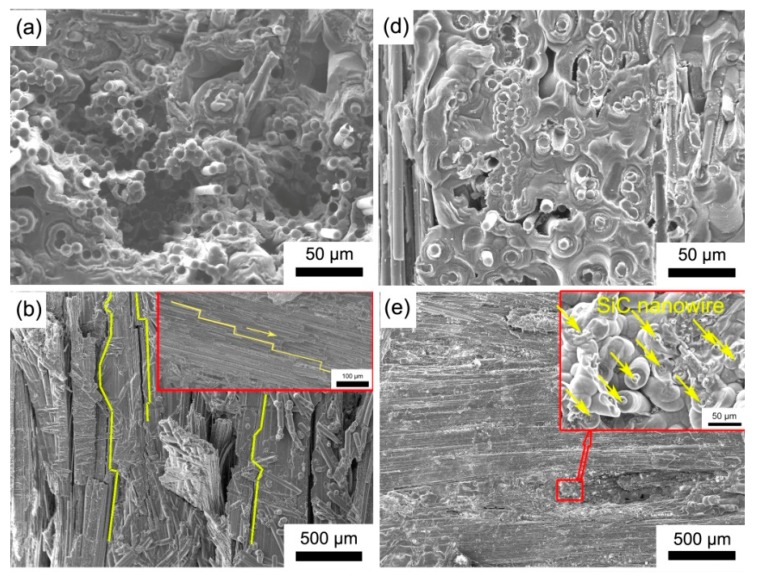

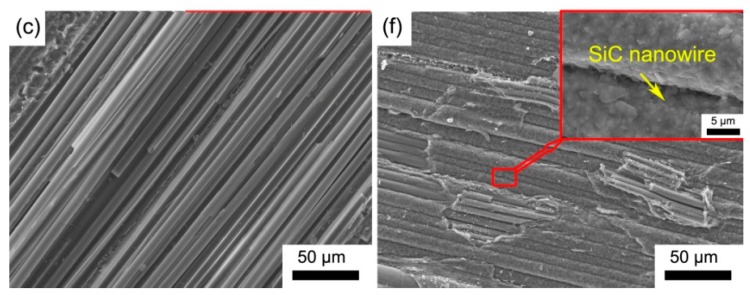
SEM images of compressive fractures and ILSS surfaces for the C/C (**a**–**c**) and SiCNW-C/C (**d**–**f**) composites.

**Figure 8:**
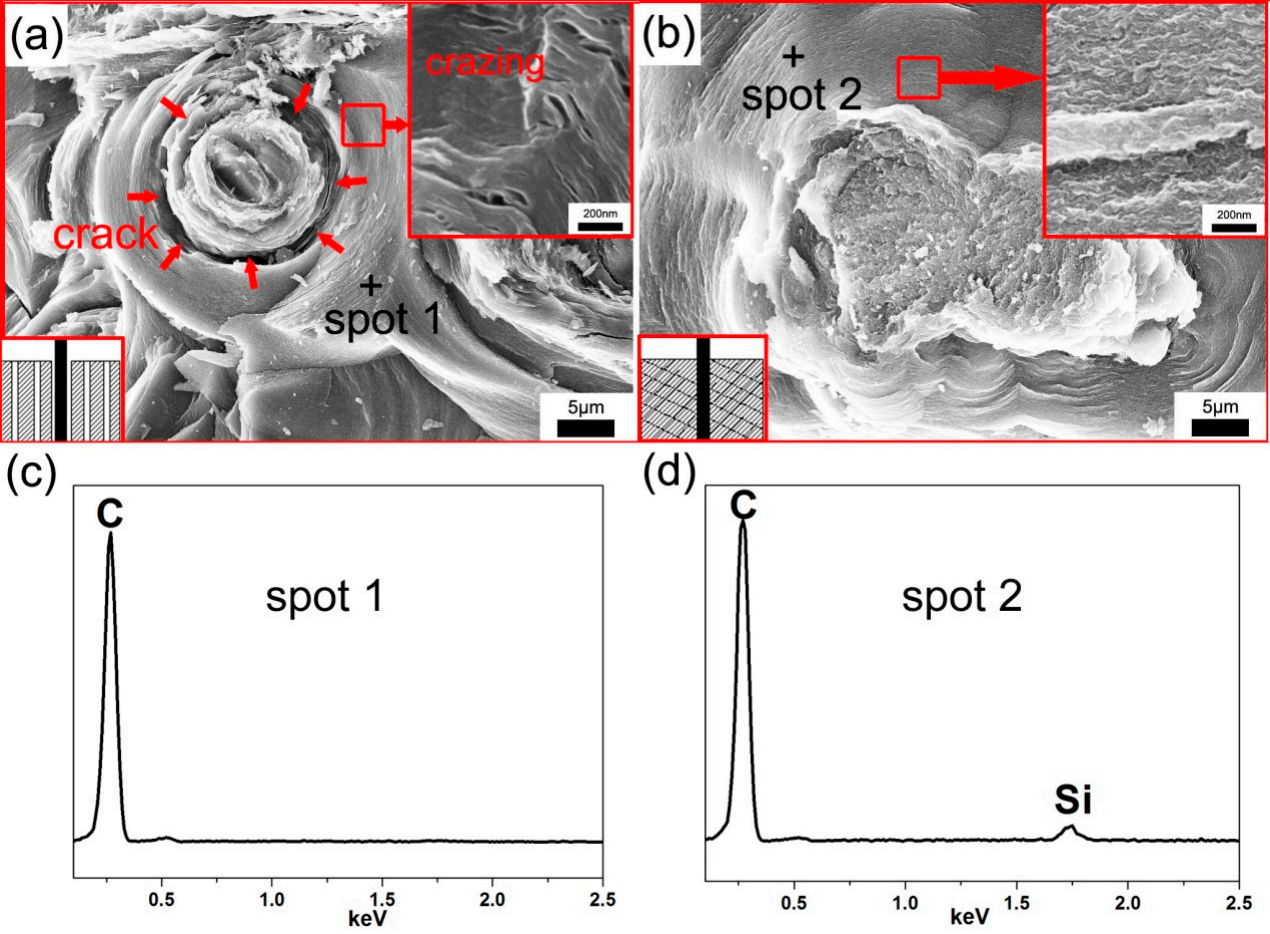
SEM images and EDS spectrums of the representative failure specimens from C/C (**a**,**c**) and SiCNW-C/C (**b**,**d**) composites, perpendicular to fiber direction.

In order to understand the reinforcement mechanism of C/C composites in in-situ grown SiCNW, the fracture surfaces of C/C and SiCNW-C/C composites were observed by SEM. Figure 7 shows the morphology of the fracture surface in C/C and SiCNW-C/C composites after compressive stress and ILSS. When the compressive stress was loaded on composite samples, the compressive strength mainly depended on the matrix cohesion [37]. In Figure 7a, the fracture surface of C/C was characterized by a rough structure accompanying bundles of fiber pull-out. As for SiCNW-C/C—shown in Figure 7d—the fracture surface was flat and nearly absent. This fracture surface could be attributed to the strongly enhanced cohesion in the matrix and also the powerful mechanical interlocking at the F/M and M/M interfaces, which allowed destructive cracks to extend into and through CFs (carbon fibers), without interfacial debonding (this has been detailed in Figure 8). In Figure 7b, the fracture surface of the C/C composites showed lots of stepwise fractured pyrolytic carbon (PyC) and many holes. The zoomed-in area in the top right corner of Figure 7b shows a step-shaped fracture along the fiber axis. These fracture steps resulted from the annular cracks that supplied paths for the spreading and linking up of destructive cracks, leading to the formation of stepwise PyC. There were no obvious stepwise fractures or holes but many SiC nanowires in the SiCNW-C/C, as shown in Figure 7e, indicating that SiCNWs could efficiently impede the propagation of destructive cracks along defects of the PyC matrix. When the interlaminar shear stress was loaded on composite samples, the shear strength mainly depended on both the matrix cohesion and F/M interfacial bonding strength. In Figure 7c, the smooth and clean fiber surface suggested a serious matrix delaminating between carbon fibers and the PyC matrix in the C/C composite, implying that primary fracture behaviors could be attributed to a typical delamination failure, which was similar to the failure mode observed in the compression test. As for the SiCNW-C/C—shown in Figure 7f—matrix delaminating was inhibited and many damaged carbon fibers could be observed in the shearing fracture surface, indicating that the interfacial strength between fibers and matrix was enhanced. In addition, faulted SiC nanowire can also be seen from the inset of Figure 7f.

SEM investigations—shown in Figure 8—into fracture surfaces of two kinds of composites, perpendicular to fiber direction, were provided to further explore SiC reinforcing mechanisms. As shown in Figure 8a, some annular cracks and small crazing (enlarged SEM image inset) could be detected easily, which implied a poor F/M and M/M interface bonding in C/C composites. Compared with C/C, the presence of SiC—shown in Figure 8b—in SiCNW-C/C composites could effectively repair this crazing and reduce annular cracks. Corresponding EDS spectrums—shown in Figure 8c,d—could also prove this. According to the analysis above, failure mechanism schematic modeling of two kinds of composites, during the loading process, was established, as shown by the inset pictures in the left corner of Figure 8a,b.

## 4. Conclusions

SiCNW-C/C composites were obtained by a combination of sol-gel and chemical vapor infiltration processes. SiCNWs were in-situ synthesized inside of the C/C composites. No catalyst was introduced in the whole preparation process, which was environmentally friendly and cost-saving. The initial oxidation decomposition temperatures of C/C, modified by SiCNWs, was reduced by about 150 °C. Mechanical property tests indicated that there was a significant increase in the out-of-plane and in-plane compressive strengths, as well as, interlaminar shear strength of the C/C composites. SiCNWs improved simultaneously the F/M and M/M interfaces. SiCNWs not only efficiently decreased defects between interfaces, but also prevented crack growth. Hence, this work might open up a possibility to produce SiC-reinforced C/C composites with excellent mechanical strength, ductility, and toughness to replace traditional C/C in industries.

## Figures and Tables

**Table 1:** Mechanical properties testing of C/C and SiCNW-C/C composites.

Samples	Out-Of-Plane Compression		In-Plane Compression		ILSS
	σ (MPa)	E (GPa)	σ (MPa)	E (GPa)	τ (MPa)
C/C	68.18	2.72	90.84	1.99	11.36
SiCNW-C/C	223.13	4.05	190.06	4.21	32.94
Increment (%)	249.3%	48.9%	109.2%	111.6%	190.0%
